# Capture stress and post-release mortality of blacktip sharks in recreational charter fisheries of the Gulf of Mexico

**DOI:** 10.1093/conphys/coaa041

**Published:** 2020-05-18

**Authors:** John A Mohan, Elizabeth R Jones, Jill M Hendon, Brett Falterman, Kevin M Boswell, Eric R Hoffmayer, R J David Wells

**Affiliations:** 1 Texas A&M University at Galveston, 1001 Texas Clipper Road, Galveston, TX 77553, USA; 2 Gulf Coast Research Laboratory, The University of Southern Mississippi, 703 East Beach Drive, Ocean Springs MS USA; 3 Louisiana Department of Wildlife & Fisheries, 2045 Lakeshore Drive, Suite 416, New Orleans, LA USA; 4 Coastlines and Oceans Division of the Institute of Environment, Florida International University Biscayne Bay Campus, 3000 NE 151st St, MSB359, North Miami, FL 33181; 5 National Marine Fisheries Service, Southeast Fisheries Science Center, Mississippi Laboratories, 3209 Frederic St., Pascagoula, MS 39567, USA; 6 Francis Marion University, Department of Biology, 4822 E. Palmetto St., Florence, SC 29506, USA

**Keywords:** blood pH, hematocrit, lactate, pop-up satellite archival transmitting tag

## Abstract

Understanding the stress responses of sharks to recreational catch and release fishing has important management and conservation implications. The blacktip shark *Carcharhinus limbatus* is a popular recreational species targeted throughout the western, central and eastern Gulf of Mexico (Gulf) yet it is unclear what levels of physiological stress result from catch-release fishing practices with hook and line gear and if the stress levels result in post-release mortality. This study correlates physiological response to stress through blood chemistry analysis and examines post-release behaviour of adult blacktip sharks caught to determine post-release mortality rates. Release behaviour was determined by pop-up satellite archival transmitting (PSAT) tags that record temperature, depth and light level data. To quantify physiological stress levels, blood samples were collected from 52 blacktip sharks and a suite of metabolic and osmotic markers were measured. Thirty-six of those blacktip sharks were also outfitted with a PSAT tag yielding time-at-large from 3 to 180 days. Of the 36 tags, 22 (61%) provided sufficient data to confirm post-release fate and 11 (31%) were recovered providing high-resolution data. Tag data suggests a post-release morality rate of 22.7% (95% confidence interval 7.8–45.4%), with mortality occurring within minutes (immediate mortality) to over 12 h post-release (delayed mortality). Compared to survivors, immediate mortalities exhibited significantly higher lactate (median 2.8 mmol/L_survivor_ vs 5.9 mmol/L_immediate mortality_) and significantly lower hematocrit (median 24.4% _survivor_ vs 14% _immediate mortality_) levels, but no difference was detected between survivors and delayed mortalities. Higher mortality in the western (30%) compared to the central (20%) Gulf may be due to shark handling. All PSATs from mortalities (*N* = 5) were recovered, and archived data revealed evidence of tag ingestion by predators. Results suggest reduced fight time, decreased handling time and limited air exposure provide blacktip sharks the best survival chances after release by recreational anglers.

## Teaser

This study examines the stress associated with recreational rod and reel capture of blacktip sharks and estimates how many sharks survive once released in the Gulf of Mexico. A post-release mortality rate of 23% was determined, with some sharks dying immediately while others experienced a delayed mortality after 12 h.

## Introduction

Sharks are popular target species of recreational anglers due to their large size and strong fighting and jumping power when captured by rod and reel (Babcock 2009). Many sharks are released after hook and line capture for regulatory or conservation purposes (Cooke & Schramm 2007), but recreational capture of sharks inflicts both physical and physiological stress (Skomal 2007). Directly linking fishing and handling practices to stress levels and understanding stress levels associated with post-release mortality are important for establishing management recommendations and promoting conservation of discarded sharks (Musyl & Gilman 2019). As recreational fishing for sharks continues to increase in popularity (Ajemian *et al*. 2016, Gibson *et al*. 2019) it is essential to quantify how catch and release practices influence shark physiology, survival and ultimately populations.

In sharks, the primary stress response initiates when the neuroendocrine system circulates corticosteroids that mobilize energy stores to maintain performance, oxygen and osmotic balance (Skomal 2007). These cascading events lead to the secondary stress responses including changes in respiration and metabolism, disturbance of acid-base balance and disruption of ionic and osmotic homeostasis (Hoffmayer & Parsons 2001, Skomal & Mandelman 2012). Blood biomarkers including pH, pCO_2_, lactate, glucose, hematocrit and osmolality can therefore be measured to quantify the overall physiological stress level of the shark. Some studies have found that stress parameters in shark blood can confidently predict post-release fate (Moyes *et al*. 2006, Hight *et al*. 2007, Marshall *et al*. 2012), while other studies do not find links between blood chemistry and post-release mortality (Whitney *et al*. 2017). It is likely that high variation in individual responses within populations and among species prevents strong predictive relationships (Gallagher *et al*. 2016). It is also challenging to eliminate handling effects, which occur as sharks are being restrained or exposed to air as blood samples are drawn that may bias blood chemistry values or post-release fate. Possibly the most difficult stress response to investigate is the tertiary response, comprising physiological changes at the organismal level that affect growth rates, reproductive output, predator avoidance and disease resistance (Skomal & Mandelman 2012). Tertiary responses manifest much later after the capture event, so connections between capture stress and mortality may be elusive.

Capture-related stress responses in sharks are species-specific (Gallagher *et al*. 2014, Jerome *et al*. 2018). Blacktip sharks (*Carcharhinus limbatus*) are particularly sensitive to capture due to intensive swimming acceleration when hooked (Brunnschweiler 2005, Gallagher *et al*. 2016). Post-release mortality estimates for blacktip sharks captured and released in recreational fisheries have been estimated using acoustic and satellite telemetry (Weber *et al*. in press) and acceleration data loggers (Whitney *et al*. 2017). Recreational post-release mortality rates for blacktip sharks range from 9.7% for sharks captured by experienced charter captains in the eastern Gulf of Mexico (Whitney *et al*. 2017) to 18.5% for blacktip sharks caught by common recreational anglers off the east coast of the USA (Weber *et al*. in press). This study quantifies physiological stress associated with conventional recreational angler rod and reel capture of blacktip sharks using blood chemistry analysis and represents the first attempt to estimate regional post-release mortality rates using pop-up satellite transmitting tags for sharks captured in the eastern, central and western Gulf of Mexico.

## Methods

### Angling, phlebotomy and tagging methods

**Figure 1:**
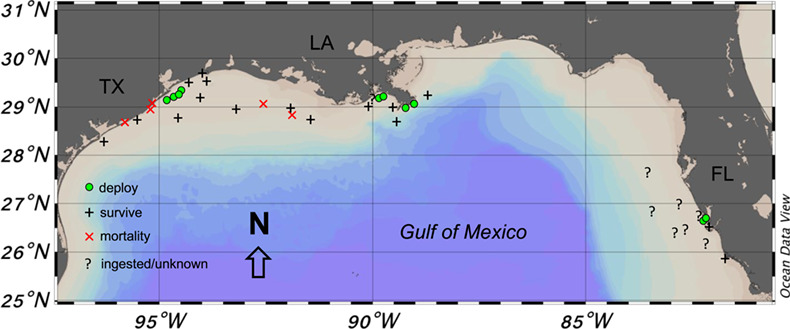
Map of PSAT tag deployment and release locations for adult blacktip sharks *Carcharhinus limbatus* in the Gulf of Mexico. Green circles indicate locations where tags were deployed, and symbols display locations were tags were first detected by Argos satellite. Symbol types also indicate the status of each individual (black cross = survive; red X = mortality;? = ingested/unknown)

Shark fishing was conducted in September and October of 2016 and 2017 in three regions of the Gulf of Mexico (Gulf) western (Texas), central (Louisiana) and eastern (Florida) (Fig. 1). Tackle and fishing techniques replicated conventional charter fishing practices that included steel leader material, either a circle or J style hook, and fresh or frozen bait. For each hooked shark, fight time was recorded as the time between hooking and landing the shark into the boat when the gills were exposed to air. The method of landing and handling the shark was conducted by the boat captain to replicate the techniques typically used in each regional fishery. In all regions, blacktip sharks were brought out of the water and on deck of the boat for the captain to take photos of the shark with the angler, with air exposures variable among individual sharks and anglers. Only in the central Gulf was a large landing net used to bring sharks on deck. In the western Gulf, a landing rope was used around the head (often around the gills) or tail and often lifted out of the water by the gills, while in the eastern Gulf the sharks were lifted by grabbing the jaw anterior to the gills. No guidance on shark handling was provided by the scientists, and captains handled the sharks using their typical methods. Once a shark was on deck, it was restrained on its side, measured for precaudal length (PCL), fork length (FL) and total length (TL), and the sex was determined. A caudal venipuncture was performed with an 18-G hypodermic needle and syringe to collect ~1–3 mL of blood, immediately after taking length and sex measurements. Blood was then transferred to vacutainers (BD Vacutainer) coated with lithium heparin to prevent coagulation. To attach the PSAT, a hole was drilled at the anterior base of the dorsal fin (~1/3 from fin origin) for the PSAT tether, and an additional hole was drilled at the posterior middle section of the dorsal fin (closer to the apex) for a plastic roto tag with a unique identification number and study contact information. The PSAT tag tether consisted of 136 kg nylon monofilament (Sufix 1.6 mm diameter) covered in a Tygon tubing sleeve (to reduce chaffing) and secured with aluminium oval crimps (1.8 mm). Two PSAT tag styles manufactured by Wildlife Computers were used: a survivorship pop-up archival tag (sPAT) and miniPAT. The sPATs were pre-programmed for 30-day deployments recording daily minimum and maximum depth temperature and light levels and set to release if they experienced constant depth (±1 m) for > 24 h. The miniPAT tags were programmed for 180-day deployments recording depth, temperature and light level every 5 min and set to release under the condition of constant depth (± 1 m) for 5 days. Once the tagging and blood collection were complete, the shark was released at the capture site, the time was recorded and the post-release condition was scored as good (rapid swimming upon release), fair (slow swimming upon release, no revival time) or poor (slow to no swimming upon release, lengthy revival time). A GPS location, water depth and water quality parameters including salinity, dissolved oxygen (mg l^−1^) and temperature (°C) were measured using a YSI Pro Plus at ~ 1-m depth at each capture

### Blood sample analysis

Immediately after releasing the shark, whole blood samples were processed on the boat. A micropipette was used to inject 95 μL of whole blood into an iSTAT cartridge (CG4+) to obtain measurements of lactate, pH and pCO_2_ on a handheld Abaxis iSTAT VetScan blood analyzer (Abott Point of Care, Princeton, NJ, USA). Because iSTAT instruments are designed for mammalian use, pH and pCO_2_ values were corrected using previously validated equations to account for lowered body temperatures in elasmobranchs (Mandelman and Skomal, 2009). An additional lactate measurement was acquired using a calibrated Nova Biomedical Lactate Plus meter and test strips (Nova Biomedical, Waltham, MA, USA), and the two lactate values were averaged. Blood samples were then placed on ice until returning to the laboratory. In the lab, hematocrit was measured by filling duplicate capillary tubes three-fourths full and spinning on an IEC Micro MB microhematocrit centrifuge for 5 min at 10 000×*g*. Hematocrit measurements were then determined using a microhematocrit capillary tube reader disk as the proportion of red blood cells to total volume. Whole blood samples were then transferred into 1.5-mL microcentrifuge tubes and centrifuged at 5000×*g* for 5 min. The plasma portion was transferred to vials by pipetting into individual 1.5-mL tubes, and both plasma and red blood cells were stored at −80°C until analyzed.

Additional blood analyses were conducted at The University of Southern Mississippi Gulf Coast Research Laboratory (GCRL). Plasma osmolality was measured with a Vapro 5520 osmometer (Wescor Environmental, Logan, UT, USA) using 10 μL of sample according to the manufacturer’s instructions. Chloride was measured with a Labconco chloridometer (Labconco, Corporation, Kansas City, MO, USA) set to low using 10 μL of plasma. The major nitrogenous osmolytes, urea and trimethylamine-N-oxide (TMAO) were quantified using a commercial assay (QuantiChrom, BioAssay Systems, Hayward, CA, USA) and a spectrophotometric assay (Wekell & Barnett 1991), respectively. Glucose measurements were determined via colorimetric assay (Cayman Chemical, Ann Arbor MI, USA). An attempt was made to measure total corticosteroids using a previously validated commercial assay; however, due to problems with the assay, this data was eliminated from further analysis.

### Data analysis

Temperature, depth and light level data from each PSAT tag were plotted and examined to determine the status of each shark. A shark was considered to survive, if depth, temperature and light level varied over time, indicating movement through the water column, which is necessary for obligate ram ventilator blacktip sharks. Constant bottom depth (>1 h) with changing light level was considered to be a mortality, as the data suggests the tag was attached to a motionless deceased shark. All of the tags that displayed mortality characteristics (constant depth with light changes) followed by changing depth with low light levels suggest evidence of tag ingestion by predators. Further confirming this was the recovery of several tags with intact tethers, indicating release mechanisms were not triggered. We believe a predator preyed on the shark and tag and later regurgitated the tag allowing it to be recovered.

To estimate error associated with the post-release mortality rate, a 95% binomial confidence interval was calculated using the Clopper-Pearson exact method (Weltersbach *et al*. 2018). Generalized linear models (GLMs) were used to explore how capture factors (fight time, handling time) and environmental factors (temperature and salinity) as independent variables related to blood physiology parameters (lactate, pH, pCO_2_, TMAO, urea, total N osmolality, osmolality, glucose and hematocrit) as dependent variables. Individual models were run using the glm package in R version 3.3.3 using a Gaussian distribution in the form: blood biomarker ~ fight time + handling time + temperature + salinity. Independent variables were explored for multicollinearity using variance inflation factors, which were all < 2, and thus all variables were retained in the models. Dependent variables were examined for normality, and only lactate had a high likelihood of coming from a lognormal distribution; thus, a log transformation was used for lactate. To determine if blood chemistry values differed between survivors, immediate mortalities and delayed mortalities, a non-parametric Kruskal–Wallis ANOVA test was performed due to low sample size with significance determined if *P* < 0.05.

## Results

**Figure 2:**
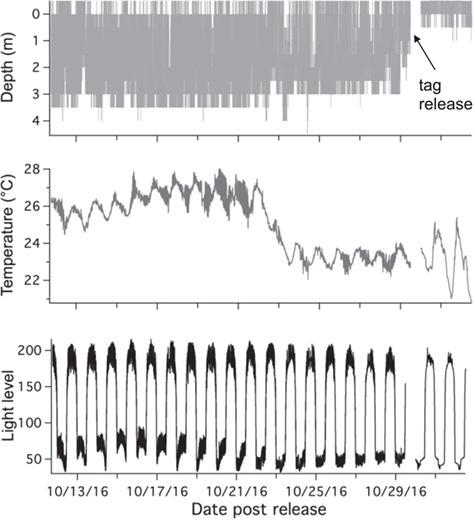
Example of PSAT tag data from a survival (FL08). Constant changes in all variables indicate tag attached and shark is moving and exhibiting survival behaviour

**Figure 3:**
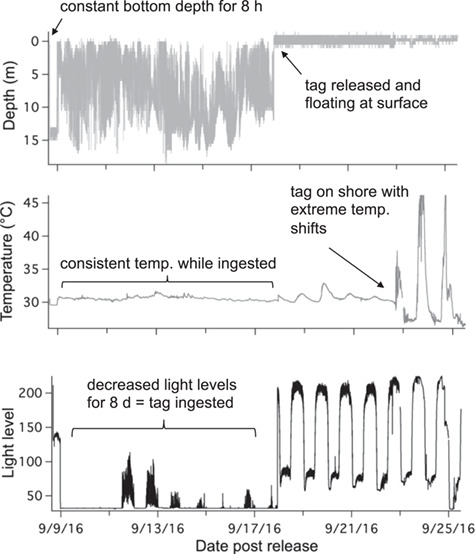
Example of PSAT tag data from an immediate mortality (TX08) showing constant depth an no movement for 8 hours, followed by tag ingestion by a predator

**Figure 4:**
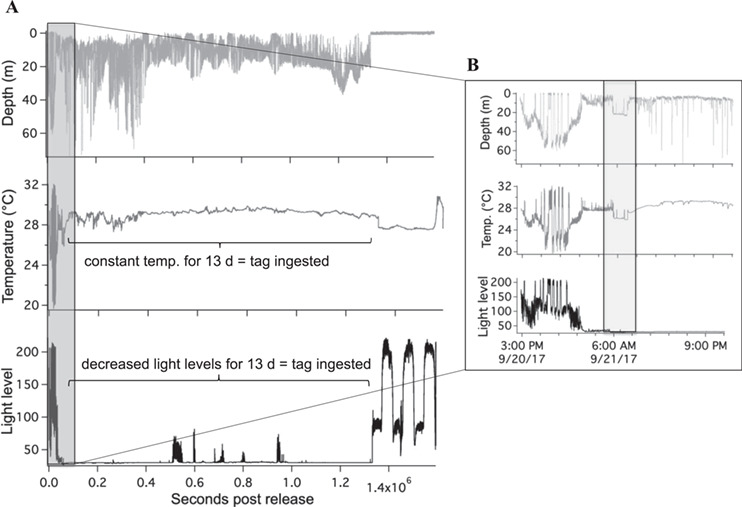
Example of tag data from delayed mortality (LA22) demonstrating full archive (**A**) and zoomed in plot of the first 14 h (**B**)

A total of 52 blacktip sharks were captured and sampled for blood. Of those sharks, 36 were also tagged with PSATs (11 sPAT; 25 miniPAT) to determine post-release behaviour with 11 deployed in the western Gulf (Texas), 13 in the central Gulf (Louisiana) and 12 in the eastern Gulf (Florida). Of the 36 tags, 14 tags did not provide conclusive data (zero or too few Argos messages transmitted or received) on shark fate leaving 22 tags (61%) to determine overall mortality; eleven tags (31%) were recovered providing high-resolution archived datasets, including *x*-, *y*- and *z*-axis accelerometer measurements. One shark was foul-hooked with a very extensive fight time (43 min) and was excluded as it was dead upon release and was considered an at-vessel mortality. A total of 17 sharks displayed vertical movements in the water column and regular changes in daily light level and temperature, indicating that the tag remained attached to the dorsal fin and the shark was exhibiting survival behaviour (Fig. 2). Three of the sharks tagged in the western Gulf exhibited immediate mortality within seconds (22 s) to minutes (1.5 min), where the sharks went to the bottom and remained motionless (constant *z* acceleration values) for many hours (7–18 h) before the tag was ingested by a predator, revealed by changing depths but low light levels and constant temperatures (Fig. 3). Two of the sharks tagged in the central Gulf exhibited vertical movements in the water column for 1.5 to 14 h, before remaining motionless on the bottom for several hours, indicating delayed mortality (Fig. 4). The tags from all five mortalities were recovered, which provided high-resolution archived data to confirm post-release fate. The percentage of tags providing conclusive post-release mortality rates for each region were 90% western Gulf, 77% central Gulf and 17% in the eastern Gulf (Table 1; Fig. 1). The overall post-release mortality rate was 22.7% (Table 1) with a binomial 95% confidence interval of 7.8 to 45.4%. The regional post-release mortality rates were 30% for the western Gulf, 20% for the central Gulf and 0% for the eastern Gulf, but low sample sizes (*N* = 2) prevent robust regional post-release mortality estimates for the eastern Gulf.

**Figure 5:**
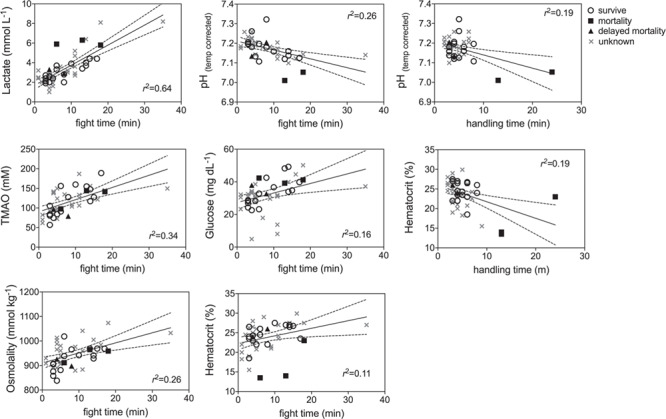
Linear regression plots of capture factors (fight time, handling time) that significantly affected blacktip blood chemistry (see Table 2 for parameter estimates) for survivors (open circle), mortalities (filled squares), delayed mortalities (filled triangle) and unknown (gray x) status.

**Figure 6:**
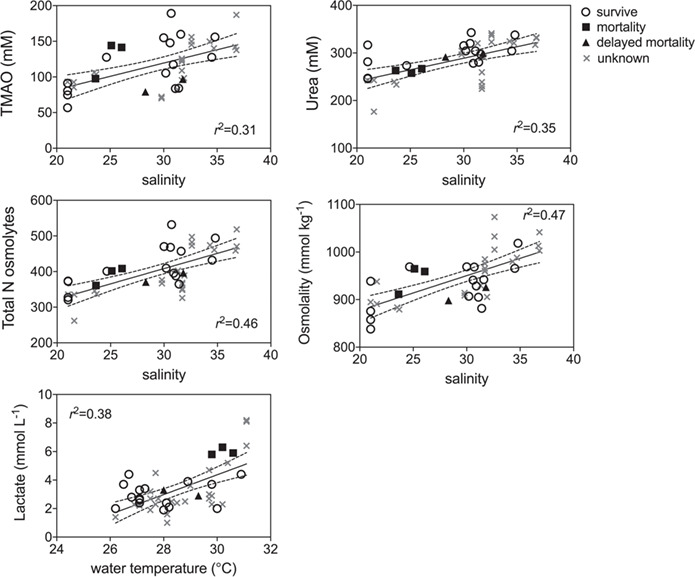
Linear regression plots of environmental factors (temperature, salinity) that significantly affected blacktip blood chemistry (see Table 2 for parameter estimates) for survivors (open circle), mortalities (filled squares), delayed mortalities (filled triangle) and unknown (gray x) status

**Figure 7:**
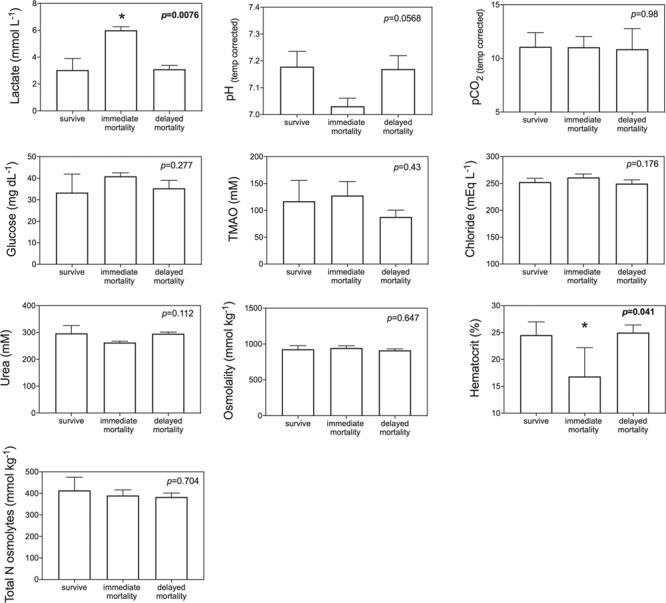
Bar plots comparing blood chemistry parameters among survivors, mortalities and delayed mortalities; Kruskal–Wallis nonparametric tests were performed, with significant *P* values in **bold** and indicated with asterisks

GLM model results indicated that blood biomarkers were significantly related to fight time, handling time, temperature and salinity (Table 2). Lactate, TMAO, osmolality, glucose and hematocrit all increased with fight time, while pH decreased with both fight time and handling time and hematocrit decreased with handling time (Fig. 5; Table 2). Blood chemistry also varied by environmental parameters, with TMAO, urea, total N osmolytes and osmolality increasing with salinity, while lactate increased with temperature (Fig. 6: Table 2). Kruskal–Wallis tests revealed that lactate (*P* = 0.0076) and hematocrit (*P* = 0.041) were significantly different among post-release groups, but no other blood chemistry parameters differed significantly among survivors, immediate mortalities or delayed mortalities (Fig. 7). Compared to survivors, immediate mortalities exhibited significantly higher lactate (median 2.8 mmol/L_survivor_ vs 5.9 mmol/L _immediate mortality_) and significantly lower hematocrit (median 24.4% _survivor_ vs 14% _immediate mortality_) levels. It must be noted however that due to an iSTAT malfunction, one pH measurement was not obtained for one of the immediate mortalities, which may have affected the non-significance of reduced pH (*P* = 0.0568) measured in immediate mortalities (Fig. 7).

## Discussion

A post-release mortality rate of 22.7% (95% binomial confidence range 7.8 to 45.4%) was determined for blacktip sharks caught and released by recreational anglers in the western, central and eastern Gulf of Mexico. Blacktip sharks displayed immediate mortalities where they perished within seconds to minutes post-release, but also delayed mortalities, where they exhibited survival behaviour for 1.5–14 h post-release prior to mortality behaviour. The post-release mortality rates were higher in the western Gulf (30%), and all three blacktip sharks exhibited immediate mortality, compared to the central Gulf (20%) where two sharks displayed delayed mortality. Blood chemistry variables were significantly related to fight time, handling time, temperature and salinity, but when comparing data from sharks with known fates, only lactate (increase) and hematocrit (decrease) were significant in blacktip sharks that showed immediate mortality.

Other studies have estimated post-release mortality rates for blacktip sharks captured by recreational hook and line (Whitney *et al*. 2017, Weber *et al*. in press). Whitney *et al*. (2017) used acceleration data loggers (ADLs) on 31 blacktip sharks captured by Florida recreational charter anglers and determined that only 9.7% died. A post-release mortality rate of 9.7% for blacktip sharks is likely a low estimate for typical recreational fisheries, as all sharks in that study were captured by experienced charter boat captains, and the sharks were kept in the water when tagging and handling. In this study, angler experience varied from novice to intermediate experience level, which represents the typical angler that would hire a charter company to catch sharks. Although the anglers with different experience levels would reel in and fight the sharks, the charter captains would handle the sharks, and thus removing sharks from the water was a common practice. In contrast, the Whitney *et al*. (2017) study used highly experienced charter captains to both fight and handle sharks, and the practice of leaving sharks in the water was common. A recent study used acoustic telemetry to compare post-release mortality rates of blacktip sharks captured in boat-based fisheries versus shore-based fisheries off the east coast of the USA (Weber *et al*. in press). The overall post-release mortality rates for recreational caught blacktip sharks was 18.5%, with slightly higher post-release mortality rates for boat captured sharks (20%) versus shore-captured (17.1%). Although Whitney *et al*. (2017) reported that hook type (J vs circle) did not affect where a shark was hooked, shark condition or recovery time, Weber *et al*. (in press) suggested that hook location was the most important factor that determined post-release mortality rates, with 50% of blacktip sharks that were hooked anywhere but the jaw (i.e. tongue, oesophagus, stomach, fin or skin), dying shortly after release. Other studies have reported that physical trauma associated with anatomical hook location can influence post-release survival (Bartholomew & Bohnsack 2005, Heberer *et al*. 2010, Kneebone *et al*. 2013).

The highest post-release mortality occurred in the western Gulf (30%), where it is common practice in boat-based fisheries to remove sharks from the water in order to remove the hook and handle the shark for photographs. In the western Gulf, recreational charter captains would often use a head rope to secure and lift the sharks from the water, with the rope tight around the gills, while others also grabbed the shark by the gills. Physical damage to this delicate respiratory organ will definitely affect the animal’s ability to respire post release (Manire *et al*. 2001). These out-of-water handling and air exposure practices likely contribute the greatest influence to increased post-release mortality rate in the western Gulf. Sharks captured in the central Gulf exhibited lower post-release mortality estimates (20%). Sharks in this region were landed using large nets, which reduced the direct contact to the gills. The 10% difference between the western and central Gulf post-release mortality rates that might be due to angler handling practices has important implications for regional fisheries management. In the eastern Gulf, only two tags provided sufficient data transmission and clear data to determine post-release fate; thus, the limited sample size prevents robust conclusions regarding eastern Gulf blacktip shark post-release mortality. In the eastern Gulf, the increased water clarity and high densities of predators (sharks and mackerels), likely resulted in depredation of the tags, causing premature tag release (Kerstetter *et al*. 2004). All five tags from confirmed mortalities were recovered and provided archived datasets indicating tag ingestion after shark mortality. Predator ingestion of electronic tags seems to be common in post-release mortality studies (Lear & Whitney 2016, Weber *et al*. in press).

Previous studies using acceleration data loggers have estimated the time it takes a shark to recover post-release based on quantified swimming metrics (Whitney *et al*. 2016, 2017). Blacktip sharks behaviourally recovered from hook and line capture within 11 ± 2.6 h (Whitney *et al*. 2017), with a range of recovery times from 5.1 to 19.5 h (Whitney *et al*. 2016). Whitney *et al*. (2017) also observed that all recreationally caught blacktip mortalities in Florida occurred within 2 h, which is similar to the results shown here of sharks dying immediately within seconds to minutes and after 1.5 h. One blacktip shark from the central Gulf survived for 14 h post release, but then experienced mortality where the shark remained motionless for 2 h before the tag exhibited signs of ingestion. Low dissolved oxygen, or hypoxia is common in the central Gulf and may have been an environmental factor contributing to the delayed mortality. Although directly attributing this delayed mortality to the capture event that occurred 14 h earlier is complex, it is within the range of previously observed behaviour recovery estimates for blacktip sharks (Whitney *et al*. 2016, 2017). Perhaps delayed mortality occurs for sharks that are unhealthy pre-capture and the capture-related stress further reduces health and survival, or the unhealthy shark is more vulnerable to predation post-release (Weber *et al*. in press), or more susceptible to environmental stress such as hypoxia.

**Table 1:** Status of blacktip sharks (*Carcharhinus limbatus*) tagged with PSATs in the western, central and eastern Gulf in 2016 and 2017

Shark_ID	Status	Region	Date capture	Fight time (min)	PCL mm	Condition	Sex	PTT	# days tagged
BT_TX_03	Mortality	West	9/7/2016	18	1180	Good	M	**163 966**	<1
BT_TX_04	Mortality	West	9/7/2016	13	1140	Fair	F	**163 971**	<1
BT_TX_05	Survival	West	9/7/2016	15	1070	Good	F	163 961	102
BT_TX_08	Mortality	West	9/8/2016	6	1050	Poor	F	**163 957**	<1
BT_TX_09	Survival	West	9/12/2017	14	1294	Fair	F	**172 297**	30
BT_TX_10	Survival	West	9/12/2017	17	1120	Fair	F	**172 300**	30
BT_TX_11	Survive_recapture	West	9/12/2017	14	1339	Good	F	171 791	78
BT_TX_12	Survival	West	9/12/2017	13	1220	Good	F	172 295	30
BT_TX_14	Survival	West	10/13/2017	10	1195	Good	M	171 796	90
BT_TX_15	Survival	West	10/18/2017	8	1180	Good	M	172 294	30
BT_TX_16	ND	West	10/18/2017	5	1060	Good	M	110 480	
BT_LA_11	ND	Central	10/17/2016	5	1010	Good	F	163 955	
BT_LA_12	ND	Central	10/17/2016	4	1073	Good	F	163 960	
BT_LA_13	Survival	Central	10/18/2016	6	1240	Fair	F	163 967	15
BT_LA_14	Survival	Central	10/18/2016	3	890	Good	M	163 963	87
BT_LA_15	Survival	Central	10/18/2016	4	1065	Fair	F	**163 969**	139
BT_LA_16	Survival	Central	10/18/2016	3	1140	Good	F	**163 958**	112
BT_LA_17	ND	Central	10/18/2016	3	1210	Good	F	163 952	
BT_LA_18	Survival	Central	9/6/2017	4	1120	Good	F	172 301	30
BT_LA_22	Delayed_mortality	Central	9/19/2017	8	1010	Fair	F	**171 795**	<1
BT_LA_24	Survival	Central	9/20/2017	3	1150	Good	F	110 478	81
BT_LA_25	Survival	Central	9/20/2017	5	1010	Good	F	172 296	30
BT_LA_26	Survival	Central	9/20/2017	3	1090	Good	F	171 790	177
BT_LA_27	Delayed_mortality	Central	9/20/2017	4	1080	Fair	F	**172 302**	<1
BT_FL_01	?	East	10/9/2016	11	1220	Poor	F	163 959	<1
BT_FL_02	?	East	10/9/2016	9	1080	Fair	M	163 968	<1
BT_FL_03	Ingested	East	10/10/2016	11	1020	Poor	F	163 965	<1
BT_FL_04	?	East	10/10/2016	9	1090	Good	F	163 962	<1
BT_FL_08	Survival	East	10/11/2016	8	1080	Good	F	**163 953**	17
BT_FL_11	Ingested	East	9/27/2017	11	1250	Fair	F	172 293	<1
BT_FL_12	?	East	9/27/2017	7*	1010	Good	F	172 292	<1
BT_FL_13	Ingested	East	9/27/2017	4	965	Good	F	172 298	<1
BT_FL_14	Ingested	East	9/27/2017	14	1130	Good	F	171 794	<1
BT_FL_15	?	East	9/27/2017	18	1120	Fair	F	171 792	<1
BT_FL_16	Ingested	East	9/27/2017	35	1100	Fair	F	172 299	<1
BT_FL_18	Survival	East	9/28/2017	6	1050	Good	F	**171 793**	153

Precaudal length = PCL; male = M; female = F. PTT # s in bold indicate recovered tags with full archived data sets; ND = no data transmitted; ? = data transmitted but unclear or too few messages to determine fate

**Table 2:** Generalized linear model results of explanatory factors (fight time, handling time, temperature, salinity) influencing blood chemistry parameters of blacktip sharks

		Factor	Estimate	Standard error	*t* value	*P* value
Log(lactate)		**(Intercept)**	−1.029	0.357	−2.877	**0.0063**
	a	**Fight time**	0.018	0.003	6.275	**<0.0001**
		Handle time	0.009	0.005	1.587	0.1202
	b	**Temperature**	0.042	0.012	3.351	**0.0017**
		Salinity	0.004	0.004	0.874	0.3867
pH		**(Intercept)**	**7.345**	**0.171**	**43.061**	**<0.0001**
	c	**Fight time**	**−0.003**	**0.001**	**−2.424**	**0.0210**
	d	**Handle time**	**−0.005**	**0.002**	**−2.116**	**0.0420**
		Temperature	−0.005	0.006	−0.794	0.4330
		Salinity	0.001	0.002	0.352	0.7270
pCO_2_		(Intercept)	6.083	5.570	1.092	0.2830
		Fight time	0.059	0.043	1.370	0.1800
		Handle time	−0.103	0.080	−1.297	0.2040
		Temperature	0.274	0.202	1.357	0.1840
		Salinity	−0.088	0.063	−1.392	0.1730
TMAO		(Intercept)	−59.308	77.281	−0.767	0.4478
	e	**Fight time**	**2.215**	**0.618**	**3.586**	**0.0010**
		Handle time	1.414	1.120	1.262	0.2151
		Temperature	1.354	2.790	0.485	0.6304
	f	**Salinity**	**3.789**	**0.872**	**4.347**	**0.0001**
Urea		(Intercept)	−9.026	113.210	−0.080	0.9369
		Fight time	0.059	0.905	0.065	0.9487
		Handle time	−0.751	1.641	−0.458	0.6500
		Temperature	6.082	4.087	1.488	0.1455
	g	**Salinity**	**4.230**	**1.277**	**3.312**	**0.0021**
Total N osmolytes		(Intercept)	−68.327	145.777	−0.469	0.6421
		Fight time	2.274	1.165	1.951	0.0588
		Handle time	0.663	2.113	0.314	0.7556
		Temperature	7.435	5.263	1.413	0.1663
	h	**Salinity**	**8.019**	**1.644**	**4.877**	**<0.0001**
Osmolality		**(Intercept)**	**513.244**	**108.817**	**4.717**	**<0.0001**
	i	**Fight time**	**2.786**	**0.870**	**3.204**	**0.0028**
		Handle time	0.550	1.577	0.349	0.7294
		Temperature	7.380	3.929	1.878	0.0684
	j	**Salinity**	**6.681**	**1.227**	**5.443**	**0.0000**
Glucose		(Intercept)	45.073	31.479	1.432	0.1608
	k	**Fight time**	**0.599**	**0.252**	**2.379**	**0.0228**
		Handle time	0.269	0.456	0.590	0.5592
		Temperature	−0.599	1.137	−0.527	0.6017
		Salinity	−0.062	0.355	−0.173	0.8632
Hematocrit		**(Intercept)**	**30.546**	**9.896**	**3.087**	**0.0038**
	l	**Fight time**	**0.226**	**0.080**	**2.844**	**0.0071**
	m	**Handle time**	**−0.393**	**0.149**	**−2.632**	**0.0122**
		Temperature	−0.347	0.354	−0.980	0.3333
		Salinity	0.115	0.116	0.992	0.3277

Letters correspond to plots in **Fig. 5** and **Fig. 6**

Physiological capture stress exhibited by blacktip sharks was quantified through blood biochemistry analysis and showed that longer fight times resulted in increased lactate, TMAO, glucose, osmolality, hematocrit and decreased blood pH. The increase in lactate and decrease in pH indicates metabolic acidosis as a result of increased physical exhaustion while hooked (Mandelman & Skomal 2009). Immediate mortalities exhibited a mean lactate value of 6.0 ± 0.3 mmol/L, which was double the value compared to survivors (3.0 ± 0.9 mmol/L) or delayed mortalities (3.1 ± 0.3 mmol/L). The increased lactate values suggest anaerobic respiration and the presence of an oxygen deficiency that may be attributed to increased handling times and air exposure. Three blacktip sharks tagged in the eastern Gulf had high lactate values above 6.0 mmol/L so it is possible they experienced post-release mortality even though the PSAT tag did not provide conclusive data on shark status. However, the three eastern Gulf blacktip sharks were also caught in the warmest water temperatures (31.1°C) which is another known stressor (Hoffmayer *et al*. 2012), so it is difficult to disentangle the effects of temperature versus fight time on blood lactate levels and how blood chemistry relates to post-release mortality. If the three unknown eastern Gulf blacktip sharks did experience mortality, the overall post-release mortality estimate would increase to 32%, which is within the binomial 95% confidence interval (7.8 to 45.4%). Blacktip sharks captured on longlines exhibit much higher blood lactate levels (range 14.82–36.8 mmol/L) due to longer times on the line (range 3–12 h) (Mandelman & Skomal 2009, Marshall *et al*. 2012). A recent study examined blacktip sharks caught in the Florida commercial shark longline fishery using 92 ADL tags and found a post-release mortality rate of 44.2 ± 8.3% and blood lactate values >50 mmol/L (Whitney 2019).

Blood hematocrit levels were significantly reduced with increased handling times, and although the correlation coefficient was weak (*r*^2^ = 0.19), immediate mortalities had significantly lower hematocrit (16.8 ± 5.3%; *P* = 0.041) compared to survivors or delayed mortalities. Handling times >10 min could be resulting in blood loss, as longer handling was sometimes required to accurately target the caudal vein to obtain the blood sample. Thus, longer handling times related to blood loss at the hooking or venipuncture location may influence hematocrit results and are not indicative of physiological stress associated with the capture event. Lower hematocrit levels would suggest that blood loss, either at the hooking location of venipuncture site, would result in hemodilution. Previous studies have found that hematocrit in sharks remains unchanged after physiological stress (Hoffmayer & Parsons 2001, Manire *et al*. 2001, Hoffmayer *et al*. 2012, Jerome *et al*. 2018), but increasing hematocrit with fight time has been reported in blacktip sharks (Weber *et al*. in press) and common thresher sharks (Heberer *et al*. 2010) caught on rod and reel.

The same sharks that had reduced hematocrit levels and experienced immediate mortalities also had the lowest blood pH values < 7.1 (although pH was missing from one mortality due to an iSTAT error). The increase in blood lactate driven by metabolic acidosis is likely contributing the decrease in blood pH, and perhaps there is a critical threshold of blood pH that blacktip sharks cannot recover from. Juvenile sand tiger sharks (*Carcharias taurus*) fully recover from blood acid–base disturbance within 12 h (pH within 6 h; lactate within 12 h) (Kneebone *et al*. 2013) and juvenile dusky sharks (*Carcharhinus obsurus*) blood pH and lactate return to pre-stress levels after 24 h (Cliff & Thurman 1984).

Blood glucose levels increased significantly with fight time, but the relationship was weak (*r*^2^ = 0.16). As exertion increases the need for mobile glucose, it is likely that glycogen stores are being converted to increase this need resulting in the rise in blood levels. However, if the glucose is being utilized by the body for energy quickly, this rise may not be easy to monitor. The condition of hyperglycaemia in response to stress is commonly found in sharks (Manire *et al*. 2001, Jerome *et al*. 2018). Muscle glycogen is depleted during rapid exercise, and the increase in blood glucose may be a restorative process where glycogen stores are released from the liver (Cliff & Thurman 1984). The relative change in blood glucose levels is dependent on initial blood glucose levels that are determined by recently ingested food (Hoffmayer & Parsons 2001). Blood osmolality also increased significantly with fight time, which may be related to water shifting out of vascular compartments in response to increased intercellular lactate in the blood (Piiper *et al*. 1972, Hoffmayer & Parsons 2001).

## Conclusions

Accurate post-release mortality rates are critical for determining total mortality estimates in stock assessment models and thus directly influence management of shark populations. Post-release mortality rate estimate of 23% for recreational caught blacktip sharks is similar to a recent meta-analysis that examined 33 studies over all gear types and found a post-release mortality rate estimate of 27% for pelagic sharks (Musyl & Gilman 2019). In this study, blacktip sharks that experienced handling times over 10 min exhibited the lowest blood pH, lowest hematocrit and immediate mortality. However, the delayed mortalities that did not exhibit physiological stress indicators, may suggest that the pre-capture health and condition of sharks may influence chances of post-release survival. Results from this study indicate that to increase the chances of post-release survival, recreationally caught sharks should be minimally handled especially avoiding the gills and kept in the water when possible.

## Funding

This work was supported by the National Oceanic and Atmospheric Administration Saltonstall-Kennedy Program (Award # NA15NMF4270352).
